# Validation of Instruments for Assessing Drug Safety Management During the Conduction of Clinical Trials

**DOI:** 10.15171/ijhpm.2017.140

**Published:** 2017-12-26

**Authors:** Yaimarelis Saumell, Olga Torres, Maritza Batista, Lizet Sánchez

**Affiliations:** ^1^Group of Health Technology Assessment, Institute of Molecular Immunology, Havana, Cuba.; ^2^Research Department, Joaquin Castillo Duany’s Hospital, Santiago de Cuba, Cuba.

**Keywords:** Validation Studies, Drug, Safety Management, Clinical Trial, Cuba

## Abstract

**Background:** The management of drug safety with the collection of reliable safety data during the conduction of clinical trials conduct is essential for the registry and marketing of products. The systematic evaluation of this process, based on objective measures, requires the application of quality instruments. This study was aimed to design and validate eight instruments through the components of quality (structure, process, and results), for characterizing and assessing the process of drug safety management, during the conduction of clinical trials.

**Methods:** The eight instruments were designed according to the international recommendations for Good Clinical Practice (GCP) and comprise a knowledge survey for professionals at the investigational sites, a satisfaction scale of internal and external clients and a satisfaction survey for patients with the treatment of the adverse events. The instruments also include a checklist to evaluate the safety management infrastructure (human, material and organizational resources) in the sponsoring center, a checklist to evaluate the same criterion at the investigational sites and three checklists that evaluate adherence to regulatory requirements of essential documents (investigator’s brochure, protocol, and informed consent form). The content validity was evaluated by Delphi method and the reliability was determined by Cronbach α test.

**Results:** All the items were valued as very adequate after the second round of the expert panel. The instruments were deemed as appropriate and understandable in the pre-test performed. All responders agreed with the options given and the accessibility of the application. Only 10% of professionals at the research sites suggested that the knowledge survey was too long. Cronbach α values between .66 and .93 were obtained.

**Conclusion:** The structure, process, and outcome framework allowed for the characterization of drug safety management during clinical trials, providing a useful approach for the promoter to systematically measure and evaluate the process. The eight instruments were deemed as reliable, feasible and easy to be used for examining drug safety management while carrying out clinical trials.

## Background

The management of clinical trials is an important element to achieve their implementation with the highest quality.^1^ Among the processes that take place during the conduct of a clinical trial, the safety management of new investigational products is a key process that begins at early stages of research in humans and spreads throughout the lifetime of the product. This process involves the collection and periodic analyses of all safety data, including non-serious adverse events and laboratory data of clinical trial patients. This process also includes the assessment and submission in time to the regulators and all other stakeholders of expedited reports of serious, unexpected and with causal relationship events. In addition, it includes the preparation and submission of annual safety update reports, signal detection, and risk management activities, including the preparation of risk management plans.^2^

Clinical trials sponsors systematically emphasize the need for a proper management of clinical trial for obtaining a successful product development,^3^ Manyof them have implemented tools such as clinical trial management systems (CTMSs) that include different kinds of applications for the drug safety data collection and evaluation (eg, the Oracle applications integrated system^4,5^ and Alas Clinica^6,7^). In addition, the Patient-Reported Outcomes version of the Common Terminology Criteria for Adverse Events (PRO-CTCAE), a measurement system developed to improve the precision and reliability of patient-reported adverse events in clinical trials.^8^ It has been developed other tools such as a screening system for the early detection and treatment of any condition based on laboratory results, which may have a possible relationship with drugs and allows feedbacks the results to the physician.^9^ Another intelligent tools have been designed and have been applied new statistical approaches for visualizing safety data, providing creative ways to present the information, assist in spotting trends and signals and enhance data review.^10,11^

However, difficulties persist when it comes to measuring quality, making it difficult to ascertain whether adequate quality is being achieved and also, to verify that processes designed to promote quality are doing so.^12^ Some gaps were identified such as inconsistencies in adverse effects reported. The adverse events are not usually pre-specified and occur their misclassification.^13^ Sometimes the way quality of safety information been processed within the surveillance function is not proper,^10^ or the promoter does not have adequate resources to achieve systematic risk assessment.^14^

On the other hand, there is a need of evaluating the effectiveness of risk communications of drug safety information to healthcare providers and the public,^14,15^ even during pre-registration development; as well as to evaluate the satisfaction of the participants in the process with the risk information they receive.^16^

Although regulatory agencies around the world and the pharmaceutical industry are taking a more comprehensive and holistic approach to safety evaluation in drug development,^17-21^ many current clinical development and drug safety systems were not designed proactively, wisely or to manage risks as required by the comprehensive list of regulations for drug safety and risk management. So, these new regulations mean that the compliance per se is a necessary prerequisite but does not control the situation itself, and it is not suitable for demonstrating the safety of products and the risk management accurately.^14^

Despite the usefulness of applying risk monitoring to safety aspects in the clinical trial, the use of objective metrics from the real clinical trials also support the sponsor level of data evaluation and they are significant tools to accelerate the improvement of the quality of clinical trials data in the industry.^22^ This requires the application of surveys, checklists and other instruments that will serve as a systematic data source. The instruments and indicators can take part of a true safety and risk management integrated system that delivers results and controls the overall risk management perception.

We do not find any specific instrument for the evaluation of the drug safety management in terms of quality during the conduct of clinical trials process. This study was performed with the aim of designing and validating some instruments to assess drug safety management, as part of a strategy to strengthen the process of obtaining safety data during the conduct of clinical trials.

## Methods

### Content Validation

The methodological sequence of three fundamental phases (preliminary, exploratory and final) was followed.^23,24^ In the preliminary phase it was formed the coordinating group,^25^ composed by the author and two experienced academic researchers on the topic studied and the methodology used.

The eight instruments were designed at this phase: a knowledge survey for professionals at the investigational sites, a satisfaction scale of internal and external clients and a satisfaction survey for patients with the treatment of the adverse events. Also include a checklist to evaluate the safety management infrastructure (human, material and organizational resources) at the sponsoring center, a checklist to evaluate the same criterion at the investigational sites and finally, three checklists that evaluate adherence to regulatory requirements of essential documents (investigator’s brochure, protocol, and informed consent form) (see Supplementary file 1, Section 1).

A framework stablished by Donabedian^26^ based on quality constructs (structure, process, and outcomes) was used for describing and measuring the process (see Figure). The initial elements for characterizing the structure and process of the safety management in the first versions of the instruments were drafted in the form of items or questions. These were defined according to the recommendations of the Guideline for Good Clinical Practice (GCP) of the International Conference of Harmonization^27^ and the Report of the Council for International Organization of Medical Sciences (CIOMS) Working Group VI.^28^

**Figure:**
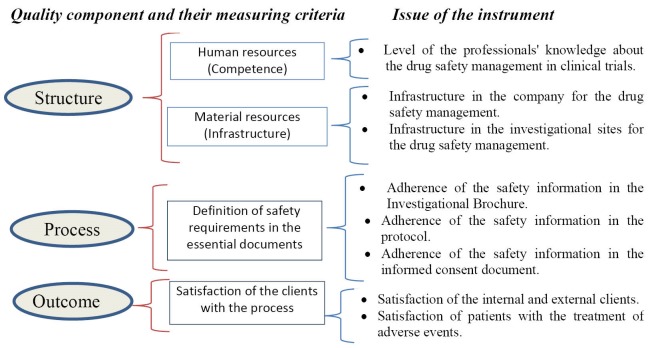
Feature Measured in Each Instrument and the Quality Component That Represent.

As a measure of the process result, it was evaluated the satisfaction of the following stakeholders: patients, investigators and co-investigators at the investigational sites, members of research ethics committees, specialist of the company’s regulatory affairs department and the regulatory agency.

Afterwards, the coordinating group carried out the analysis, discussion, and qualitative adjustment to achieve the first version.

To the end of this phase, an intentional sampling was used for a possible expert selection that would integrate a panel for the evaluation of the content validity of the instruments. According to the methodological needs and peculiarities of each instrument, it was an imperative the selection of professionals having the following criteria:

- To work as a clinician, nurse, researching manager, clinical trial monitor, or specialist of the national drug regulatory agency.
- To have five or more years of experience related to safety management in pre-registration clinical research.
- To have completed a course on clinical safety, GCP or clinical trials.

Twenty-five professionals were invited to participate. In October 2013, a self-appraisal questionnaire was sent to professionals (see Supplementary file 1, Section 2) with the purpose to collect self-assessment on each of their competence in the sources of discussion that supported this approach, using e-mail as a means of distribution and collection. From the questionnaire results, the level of competence of each expert was determined following the methodology employed by Hurtado^29^:

1- The level of knowledge was measured on a scale of 11 categories (0 to 10, where 0 represents the absence of knowledge on the subject treated and 10, full knowledge). The candidate has to tick his level of knowledge somewhere on the scale, according to his own self-assessment. The knowledge coefficient (Kc) was formed multiplying the level of knowledge by 0.1.

2- It was estimated the coefficient of argumentation (Ka) of each expert from the analysis that he makes of the sources that allowed him to argue his criteria. For this, each respondent was asked to indicate in an ordinal scale of three categories (high, medium, or low level) the degree of influence they have had in their level of competence reached each of the following sources of argument:

- Theoretical analyzes linked to the safety management during the conduct of clinical trials.
- Experience gained in the practice regarding safety management.
- Reviewed works of national authors on the subject.
- Reviewed works by international authors on the subject.
- Knowledge of the state of safety management abroad.

To assign numerical values to each source of argument according to the degree of influence were attributed the values of the standard table used by Hurtado.^29^ The number of points obtained in total, corresponding to the value of the argumentation coefficient, was calculated by the following formula:

$$\text{Ka =}\sum n_{i}=(n_{1}+n_{2}+n_{3}+n_{4}+n_{5}+n_{6}),$$

where Ka: Argumentation coefficient, n_i_: Value corresponding to the source of argument *i* (1 to 6).

3- Based on the information of the questionnaires, the competition coefficient (K) is determined using the following formula:

$K=\frac{K_{c}=K_{a}}{2}$Were considered:

High-level of competence: 0.8 ≤ K ≤ 1

Medium level of competence: 0.5 ≤ K ≤ 0.8

Low-level of competence: K < 0.5.

Exploratory phase (November-December 2013): the coordinating group e-mailed to the expert members of the panel the first version of the instruments and the corresponding validation guide (see Supplementary file 1, Section 3), with the purpose to obtain their opinion about the usefulness of the proposals concerning the quality of its performance and the effectiveness that this first application could present.

The validation guides were organized as a single ordinal scale Likert type,^30^ in which appropriateness must designate a scale from one to five (one represents a maximum full disagreement while five, shows full agreement), and the response categories were described by using the following linguistic qualifiers:

5) Very adequate (VA): The item or question wish optimally reflects the theme, concept or specific content that it tries to measure. It can provide the total grasping of that content for clarity in writing (comprehension) and adequacy of the response options (for knowledge and satisfaction surveys).

4) Quite adequate (QA): The item or question that expresses in a quite high degree, the theme, or specific content that it tries to measure. Although it requires some drafting modification is an adequate response option and provide a high level acquisition of the content.

3) Adequate (A): The item or question that takes into account an important part of the qualities of the subject or specific item that it tries to measure. Although it can be improved by the experts modifying some part of the text.

2) Little adequate (LA): The item or question that reflects a low level of adjusted theme. The concept or specific content that it tries to measure must be considered. This category involves a low adequate level of the evaluated subject. The text may be modified from the expert point of view.

1) Inadequate (I): The aspect with marked limitations and contradictions. It is that one which not allowed expressing the essential qualities of the concept or specific content item. It is intended to measure, but ideas are not stated correctly. It implies the failure of grasping or understanding the element in question and, consequently, brings about the deletion of the item.

In all the items, the experts might include additional free observations, assess the number of questions or extension of the items, and suggest the modification or, when necessary, make proposals for new items.

The statistical and quantitative analyses of the content validation for the proposals generated in previous phase were performed using the Delphi method,^31,32^ and a manual version of the Torgerson mathematical model according to Moráguez,^33^ converting the ordinal scale into interval scale (from qualitative to quantitative) with the intention to provide objectivity to the experts’ judgment. The steps are:

1. To obtain the observed frequency
2. To obtain the accumulated frequency
3. To obtain the relative accumulated frequency
4. To allocate the value of the image corresponding to each obtained relative cumulative frequency from the Z-table of the normal distribution
5. To obtain the points through the calculation of N-AR where:

$$N=\frac{Summation\;of\;Sum\;by\;Aspects}{\left(No\;of\;Valuation\;Ranges\;x\;Number\;of\;Aspects\right)}$$

and AR = Average value for each item or question.

The line is divided by categories from the cut-off and the N-P points are located to determine the category of each aspect.

$$Cut-off\;=\frac{Summation\;of\;Sum\;by\;Aspects}{No.\;of\;Aspects\;to\;Evaluate}$$

The cut-offs obtained for each category of evaluation (VA, QA, A, LA or I) determined the reach of their range limit. If the subtraction of the limit value (N) minus the average value of the item (AR) is less than the cutoff of the category then the final evaluation for this item or question is the same category. If the subtraction of the limit value (N) minus the average value of the item (AR) is greater than the cutoff of the category then the final evaluation for this item or question is the next category. At the end of the final round, the level of consensus (C) was reached for each item or question, by the expression:

$$C=\left[1-\left(\frac{V_{n}}{V_{t}}\right)\right]\times 100$$

Where *V*_n_ = Negative votes, *V*_t_ = Total votes_ ._

Decision rule: If C ≥ 75%, it means that there is a consensus among experts, but if it otherwise, (C <75%), another round of consultation should be held up to reach an agreement.^33^

Considering the modifications, adaptations or clarifications of the experts, a new proposal of each instrument was designed after the first round.

Final phase: a summary with all responses was sent to the panel members as a feedback measure and they were asked to newly complete the questionnaire validation as well as giving their opinions, regarding the ones that differed from the rest. The level of consensus is determined for each instrument after each round, and so forth until arriving at a consensus.

### Pre-test

A pre-test was carried out to each instrument with the objective of detecting interpretation errors and clearing up the lists of items or questions. The responders were asked to show their agreement with the drafting and understanding of the item, adequacy of the response options, application easiness and the extension of each instrument.

Previously, the persons responsible for the application of the instruments were instructed and trained.

The number of participants in the pre-test were the following: twenty professionals of the investigational sites for the knowledge survey, five patients, three internal and two external clients for the satisfaction scales, two professionals of the sponsor center and five of the investigational sites for the safety management infrastructure checklists, and three professionals for the checklists that assess adherence to regulatory requirements of the investigator brochures, protocols and informed consent documents respectively.

### Evaluation of Reliability

Cronbach α internal consistency coefficient was determined for the evaluation of the reliability of all the instruments. The interpretation should be that the more the value approach to 1, the greater will be the reliability. Almost all the alfa values over .70 are considered acceptable and over .80, good values.^30,32^

## Results

### Content Validation

Twenty-five professionals were invited to form the panel of experts, but only seventeen agreed to participate. As result of the evaluation of the level of competence, eight of the professionals with a high level of competence were selected, and two with the highest score in the category of the medium level (see Table).

**Table:** Results of the Self-assessment Survey for Determining the Performance Coefficient of Experts

	**E1**	**E2**	**E3**	**E4**	**E5**	**E6**	**E7**	**E8**	**E9**	**E10**	**E11**	**E12**	**E13**	** E14**	**E15**	**E16**	**E17**
K	0.85	0.80	0.45	0.95	0.60	0.95	0.70	0.70	0.55	0.80	0.65	0.95	0.55	0.55	0.90	0.65	0.80
H	x	x		x		x				x		x			x		x
M					x		x	x	x		x		x	x		x	
L			x														

Abbreviations: E_n_, expert; K, competence coefficient; H, high level of competence; M, medium; L, low level of competence.

The results of the content validation for the first five instruments (the knowledge survey, the satisfaction scale of internal and external clients, the satisfaction scale of patients, the checklist to evaluate a safety management infrastructure at a sponsor center, and the checklist to evaluate a safety management infrastructure at the investigational sites), are shown in Supplementary file 1, Section 4.

The cutoffs determined the reach of their range limit for each category of evaluation. In all the cases, the subtraction of the limit value (N) minus the average value of the item (AR) is less than the value of the respective cutoff.

Finally, after the analyses of this round, the items and questions fall into the category of “very adequate,” with a high degree of relevance since there were no negative votes.

Anyway, clarification in respect to items that are adequate response options, but which require some drafting modification, were considered. The instruments were resent to the experts for a second round and all of them maintained stability in their answers.

After the second round, 100% of the consulted experts maintained stability of their approaches valuing the questions of the questionnaire like very adequate. They considered that the structure of questions or items that compose all the questionnaires is enough for the investigation. Then, it was not necessary to carry out a new round.

For the other three instruments (checklists that evaluate adherence to regulatory requirements of essential documents), all the items were qualified as very adequate. That is why it was not necessary to represent the application of the Delphi method.

### Pre-test

The instruments validated by the panel of experts were considered appropriate and comprehensible in pre-test. All the responders were agreed with the response options, the application easiness and the extent of each instrument. No one suggested modifications.

### Evaluation of Reliability

Respect the reliability for the knowledge survey was obtained α = .7124, a value of α = .7251 for the satisfaction survey of internal and external clients, α = .9336 for the patient satisfaction survey, α = .9023 for the infrastructure checklist in the sponsoring centre and .8493 for the infrastructure checklist in the sites. For checklists that assess adherence of investigator brochure, protocols and informed consent documents to the regulatory requirements, were obtained α values of .6632, .7060, and .7025 respectively.

## Discussion

This study ensures the validity and reliability of eight instruments that will be the base of indicators for the systematic evaluation of the drug safety management at the Molecular Immunology Center. It is a part of a strategy for strengthening and complementing the global management of clinical trials.

Several authors agree with the need to design and validate new measuring instruments when there is no other one capable to measure what is needed.^30,34^ The framework of structure, process, and outcome for the organization of quality metrics was useful in defining drug safety management during clinical trials so that it can be measured and evaluated according to GCP requirements.

In a case study^12^ conducted by Pfizer and Avoca groups with the goal of implementing a framework for the oversight and analysis of Pfizer’s overall clinical trial quality, they defined outcome, predictor and contributor metrics. Although they established seven major categories for the quality outcomes and for all of them defined their outcome components. The results of the implementation of this approach did not take part of this manuscript. The majority of the aspects considered in the study mentioned in respect to the clinical trial safety management were taken into account in our study.

The designed knowledge survey will contribute to assessing systematically the level of training required by clinical trial participants for the proper identification and management of adverse events at the research site. This will help reduce the inconsistencies in adverse event classification and report in clinical trials.

The role of effective training for professional develops and obtains adequate knowledge in a highly specialized field of clinical research is as imperative as to define the various levels of training required for these professionals.^35^ To assure the investigator’s sensitivity to this point is one of the key responsibilities of the sponsor, which includes proper training of the investigative site personnel regarding the use of the relevant diagnostic terms for consistent data collection and reporting.^28^

All the checklists designed will help to control the management of the process in a systematic way, both in the context of the sponsor and in the research sites.

The use of GCP compliance checklists also supports to put plans into action to prevent future noncompliance in identified areas. Checklist results often suggest the potential root cause of noncompliance. Once the root cause is identified, clinical operations can develop corrective actions to remedy immediate issues, and design preventative actions to avoid future noncompliance in the identified area.^36,37^

The designed satisfaction surveys will contribute to the systematic evaluation of the perception of those involved in the process. We introduce the satisfaction of the different stakeholders with the process as a very important outcome because the systematic perception of each implicated can help to estimate the performance in several manners, facilitating to reach a proactive risk and safety management.^17^ The manner in which safety experiences are currently elicited during discussions with patients by the investigator and his/her staff during visits or at other times is one of the most important issues that is rarely addressed.^28^ On the other hand, the communication between the pharmaceutical industry and regulator on potential safety concerns must be frequent and systematic.^14,16^ Nelson mentioned the patient satisfaction as a quality metric for a clinical trial performance improvement since a holistic perspective.^37^

There are different criteria regarding the definition of the number of experts needed for the panel, but the majority recommends that the number of experts should range from 5 to 15 members, that should possess experience in the treated topic and independence in the evaluation approaches.^30,33,38^

In respect to the approaches of quantitative analysis of expert answers, besides completing the punctuations proposed for the estimate of the items, the qualitative contributions improved the validation process. This result is common when the Delphi method is applied.^30,38,39^

The qualification of very adequate was expected for the items of the three checklists that evaluate adherence to regulatory requirements of essential documents since these items were elaborated taken into account the recommendations of the international current regulations. The fact that, after the second round, all the items or questions of each instrument were qualified as VA, QA or adequate, suggests a high percentage of agreement among the evaluators.

Cronbach Alpha test is the most widely used method for the analysis of reliability when expressing to what extent the answers are related to each other, or measuring the same things it can also be added to a unique total punctuation.^30,32^

The internal consistency was good for the satisfaction survey of patients, internal and external clients, as well as the checklist for infrastructure in the research center and at the investigational sites. The knowledge survey was acceptable, just like the checklists that assess adherence to protocols and informed consent documents to regulatory requirements. While the checklist that assesses adherence of investigator brochure to regulatory requirements reached a value discreetly below the acceptable limit of reliability.

These instruments were validated in real trials. From each one, we are designing indicators and standards that will provide a performance measure of the drug safety management. We are working on the implementation of a continuous improvement strategy related to that, but the results are not discussed here.

The instruments could be used as part of any drug safety management system, despite the application of technological supports, the company size, and geography. We suggest their application for the diagnosis of the state of the system the first time. Then, the instruments related to the structure of the process must be applied at least once a year, and every three months those instruments for the process and outcome features.

Another aspect of the drug safety management process such as the time to send the expedited report from the site to the sponsor and from this to the regulatory agency and the adequate description and completeness of the document, need to be measured. That is the limitation of the study, and the fact that another validation as intra or interobserver reproducibility was not addressed.

## Conclusion

The structure, process and outcome framework allowed the characterization of drug safety management during clinical trials, providing a useful approach for the promoter to systematically measure and evaluate the process. The eight instruments proposed in this study are reliable, feasible and easy to use for examining drug safety management during the clinical trials conduct.

## Acknowledgements

We especially thank Ana Rosa Valls Hung, Leticia Cabrera Benítez, Meylan Cepeda Portales, and Delmis Mayra Batista Ramírez for their assistance in this study. They carried out the application of the instruments during pre-test.

## Ethical issues

The scientific committee of the investigational sites were notified, and were obtained the verbal consent of professionals and the study participants. The confidentiality of the information and its exclusive use for scientific purposes was guaranteed.

## Competing interests

Authors declare that they have no competing interests.

## Authors’ contributions

YS was responsible for the study conception and design, the data collection and analysis, and the writing of the manuscript. OT participated in the conception of the study and the critical revision of the manuscript. MB participated in the drafting of the manuscript, data interpretation and critical revision. LS participated in the statistical analysis and critical revision of the manuscript.

## Authors’ affiliations

^1^Group of Health Technology Assessment, Institute of Molecular Immunology, Havana, Cuba. ^2^Research Department, Joaquin Castillo Duany’s Hospital, Santiago de Cuba, Cuba.

## Supplementary files


Supplementary file 1 contains sections 1-4.

## Key messages

**Implications for policy makers**

- To decide if a new drug can be included in a therapeutic guide, the policy-makers need reliable safety data.
- Given the strong commitment of the pharmaceutical industry to drug and patient safety, while conducting clinical trials, it must provide policy-makers with evidence to prove that safety management in these investigations is adequate.
- The pharmaceutical sponsors can strengthen the drug safety management process taking into account the perception of all the clinical trials stakeholders.
- To increase the reliability of safety data of a new drug, all the instruments used for measuring the quality of safety management during clinical trials, need to be validated, even those based on the current regulatory norms.
- As the emphasis is on transparency, applicability, and communication, this approach to assess drug safety management should maximize the impact of these data to all stakeholders and decision-makers.

**Implications for the public**

The application of the instruments validated in this study helps to strengthen the process of obtaining drug safety data in clinical trials. The greater the number and quality of the information of the investigational drug safety obtained in a clinical trial, the lesser is the likelihood that serious adverse drug reactions will occur after the commercialization. Thus, the population will use the drug with a higher level of confidence, and clinicians will have more information about the drug safety profile. All of these contribute to guarantee the patient’s safety.
