# Supplementary material for: Validation of Instruments for Assessing Drug Safety Management During the Conduction of Clinical Trials
Source: Int J Health Policy Manag. 2017 Dec 26;7(7):623–9. doi: 10.15171/ijhpm.2017.140 (PMC6037497; doi:10.15171/ijhpm.2017.140)
Supplement: Supplementary file 1 — contains sections 1-4. [file ijhpm-7-623-s001.pdf]

Section 1

Knowledge's survey about safety management in clinical trials (for professionals at the investigational sites).

The present survey was designed regarding the recommendations of the Guideline for Good Clinical Practice (GCP) of the International Conference of Harmonization and the Report of the Council for International Organization of Medical Sciences (CIOMS) Working Group VI in respect to the management of adverse events during the development of clinical trials.
It is a part of an investigation that aims to assess -in an integral way- the process of drug safety management in pre-commercialization clinical trials.
We request your support for the development of such survey.

Thank you

Indicate the role you have played in clinical trials:
___ Principal investigator, responsible or co-investigator
___ Clinical Research Coordinator
___ Clinical Pharmacist
___ Clinical Laboratory Researcher
___ Clinical nurse

I. Given the following statements regarding safety management while conducting a clinical trial at the research site, mark true (V) or false (F) as deemed.

___ 1. An adverse event can be any unfavorable and involuntary sign, or symptom of a disease temporarily associated with the use of a medicinal product that may or may not be related to the research product.

___ 2. In order to identify the adverse event, in addition to making a detailed description, the investigator may use any definition he considers appropriate to name the event.

___ 3. Exacerbation of a pre-existing condition or symptoms should not be considered an adverse event.

___ 4. Clinical laboratory tests are an important source for the identification of adverse events during the conduct of a clinical trial with a new product.

___ 5. The availability of the crash cart and the emergency stock is essential only in Phase I clinical trials.

___ 6. If a patient leaves the clinical trial for a serious adverse event, from that moment it is not necessary to follow the outcome of the event.

___ 7. The "severity" of the adverse event refers to the intensity with which the event appears, being able to be mild, moderate or severe.

___ 8. An adverse event is classified as serious when it causes death; Threatens the life of the patient at the very moment in which it occurs; Requires or prolongs hospitalization; Causes disability, persistent or significant disability, or a congenital defect; Or constitutes a medically significant event.

___ 9. In the algorithms to determine a causal relationship with a product under investigation in the event of an adverse event, the categories of "Very likely," "Probable" or "Possible" are those that confirm that the event is related.

___ 10. The classification of the adverse event according to previous knowledge (expected or unexpected) is made prior to the analysis of the existence of a causal relationship with the product under investigation.

___ 11. The investigator should promptly inform the CEIC about deviations or changes in the protocol in order to avoid immediate risks to subjects, changes that increase the risk to subjects, any adverse drug reactions that are serious and unexpected; Or new information that may adversely affect the safety of subjects.

___ 12. In blind trials, whereby a serious adverse event the investigator opens the product code under investigation, he must document and explain to the developer promptly; and if not necessary, it does not break the blinding of the people who continue in the study.

II. Read carefully and mark a single answer option (the only one that you consider correct or complete) for each question:

13. The person responsible for identifying the adverse event is:

___ The clinical investigator
___ The study monitor
___ The Clinical Research Coordinator
___ The clinical nurse
___ The clinical pharmacist
___ The clinical laboratory researcher

14. An adverse event is considered unexpected when:

___ their nature, severity or frequency is not consistent with the information available on the product.

___ it is not expected according to the mechanism of action of the research product, the clinical status and the patient's personal pathological history.

15. In describing the adverse event in medical evolution, the following classification order must be followed:

___ Intensity → Previous knowledge → Causality → Seriousness
___ Seriousness → Intensity → Causality → Previous knowledge
___ Intensity → Seriousness → Causality → Previous knowledge

16. The regulatory agency must be notified within 72 hours of receipt by the sponsor the adverse events:

___ Severe and unexpected
___ Serious, attributed causality and unexpected

___ All serious

17. Serious and unexpected adverse events should be reported to regulatory agency within:
    ___ 72 hours from the moment the Promoter receives it.
    ___ 72 hours from the time of the event.

18 The expedited report conveying the events:
    ___ Severe and unexpected
    ___ Serious, with attributed causality and unexpected
    ___ All serious

19. The model of the report of serious (serious) and unexpected adverse events with a causal relationship and the attached documentation regarding the adverse event presented must be submitted to CECMED:
    ___ Immediately
    ___ Sooner and never after 7 calendar days if the adverse event is fatal or compromises the life of the subject, otherwise, the reporting time will be 15 days.

20. The expedited report must follow the trajectory:
    ___ Research site ⇨ Ethics Committee ⇨ Sponsor ⇨ Regulatory Agency
    ___ Research site ⇨ Sponsor and Ethics Committee ⇨ Regulatory Agency
    ___ Research site ⇨ Regulatory Agency
    ___ Research site ⇨ Sponsor ⇨ Regulatory Agency

Satisfaction’s survey for internal and external clients.

The present survey is part of an investigation that aims to assess -in an integral way- the process of drug safety management in pre-commercialization clinical trials. We request your support for the development of such survey.

The role that plays in clinical trials with CIM products:
___ Specialist of the Regulatory Agency
___ Sponsor researcher manager
___ Clinical Research Monitor
___ Clinical Research Assistant

The following statements constitute views on the management of safety information at clinical research sites during the conduct of clinical trials. Please read carefully and mark with a cross (x) the option that represents your degree of agreement with each affirmation.

**Affirmations**	**5- Strongly agree**	**4- Agree**	**3- Neither agree nor disagree**	**2- Disagree**	**1- Strongly disagre**
1. The term used for the name of the AE in medical records, ER or notification is standardized.					
2. The description of AE in medical records, models of notification or ER is appropriate.					
3. The classification of AE according to the intensity complies with the requirements of the protocols.					
4. The categories used in the classification of the AE is according to the causality algorithms proposed in the protocol.					
5. The reporting of AE in the notification models is complete.					
6. The AE information in the notification models is timely (arrives in the set time).					
7. The report of AE in the ER models is complete.					
8. The adverse event information models ER is timely (arrives in the set time).					

Satisfaction survey for patients included in clinical trials.

The present survey is part of an investigation that aims to assess the satisfaction of subjects with the attention received when appearing and adverse event during your participation in a clinical trial. We request your support for the development of such survey.

Thank you

The following affirmations assess your degree of agreement with different aspects of the attention received when appearing an adverse event that requires treatment during clinical trials. Please read carefully and mark with a cross (x) the option that represents your degree of agreement with each affirmation.

Affirmations	5- Strongly agree	4- Agree	3- Neither agree nor disagree	2- Disagree	1- Strongly disagree
1. The attention was immediate to the appearance of the AE.					
2. The doctor explained the consequences of the event for your current health status.					
3. The classification of AE according to the intensity complies with the requirements of the protocols.					
4. They explained the consequences of the AE to continue in the study and receive the product.<br>algorithms proposed in the protocol.					
5. You are satisfied with the care received when appeared and adverse event that required treatment.					
6. You were informed of the reactions that may occur after administration even before the first dose of the product.					
7. Even before the first dose of the product, you know the person to contact if you a life-threatening AE or the required/prolonged hospitalization.					
8. You are satisfied with the information received regarding the safety of the product under investigation.					

Checklist for the assessment of the infrastructure for safety management at the sponsoring center.

The present checklist is part of an investigation that aims to assess the infrastructure (human, material and organizational resources) of the clinical trials sponsor for the drug safety management. All aspects considered were defined according to the recommendations of the Guideline for Good Clinical Practice (GCP) of the International Conference of Harmonization** and the Report of the Council for International Organization of Medical Sciences (CIOMS) Working Group VI. ***
We request your support for this.

Thank you

Please, mark with a cross (X) if there is compliance, non-compliance or partial compliance.

**Features**	**Compliance***		
	**Yes**	**PC**	**No**
1. There is a database for managing and archiving the safety information.			
2. There is a register for controlling the input and output notification / ER form in the site.			

3. There is a SOP or instruction to guide the global management of safety data.			
4. Clinical safety events are analyzed (identification, coding, quantification and evaluation) as is established by pharmacovigilance.			
5. The number of notification or expedited report is controlled by investigational product or indication.			
6. The date of start and end of the AE is controlled.			
7. The dates of receipt the notification / ER of an AE are controlled.			
8. The causality of case reports and series of individual cases are evaluated.			
9. Criteria for assessing the quality of notifications or accelerated reports are defined.			
10. The evidence of communications between researchers and promoters about adverse events are archived.			
11. The results of safety corrective and preventive actions (CAPA) generated in monitoring, audits and inspections reports) are filed, followed and disseminated.			
12. The corrective and preventive actions generated of the safety CAPA are documented.			
13. The inter-institutional or informational communications about safety are sent to the stakeholders and are archived.			
14. The responses to requests for notifiers and the regulatory agency are managed and are filed.			
15. The changes in benefit/ risk for investigational product are evaluated and are communicated to researchers.			
16. Training and counseling processes about safety are coordinated for the members of the research teams.			
17. The dates of the Development Safety Update Report (DSUR) actualization are controlled.			
18. The date of update safety data in Investigator Brochure, Protocol and Informed Consent, are controlled.			
19. The risk management plans for the investigational product are elaborated.			
20. The internal audits of safety management are performed.			

*To specify bellow the cause of non-compliance or partial compliance (PC).

**International Conference on Harmonisation. E6 (R1)- Good Clinical Practice: consolidated guidelines, Step 5. May 1996. Available from: https://www.ich.org/fileadmin/Public_Web_Site/ICH_Products/Guidelines/Efficacy/E6/E6_R1_Guideline.pdf
***Council for International Organizations of Medical Sciences. Working Group VI. Geneva: CIOMS, 2005. Available from: https://cioms.ch/wp-content/uploads/2017/01/Mgment_Safety_Info.pdf

Checklist for the assessment of the infrastructure for safety management at the investigational sites

The present checklist is part of an investigation that aims to assess the infrastructure (human, material and organizational resources) for the drug safety management at the investigational sites during clinical trials conduct. All aspects considered were defined according to the recommendations of the Guideline for Good Clinical Practice (GCP) of the International Conference of Harmonization** and the Report of the Council for International Organization of Medical Sciences (CIOMS) Working Group VI. ***
We request your support for this.

Thank you

Mark with a cross (X) if there is compliance, non-compliance or partial compliance.

**Features**	**Compliance***		
	**Yes**	**PC**	**No**
1. They have a crash cart during the administration of a new investigational product.<br>1.			
2. The use of a truck stop for clinical research is documented.			
3. They have a stock of medicines for emergencies during the clinical trials.			
4. The use of emergency stock for clinical research is documented.			
5. There is a standard operating procedure or instruction for management safety data at the investigational site.			
6. All SAE are analyzed.			
7. The number of notifications and ER is controlled by investigational product or indication.			
8. The dates of start and end of the AE are controlled.			
9. The date of shipment of AE notification and ER are controlled.			
10. If necessary, the promoter is alert when signals are generated and causality series are evaluated.			
11. The quality of notifications and ER that are sent to the promoter are guaranteed.			
12. All the communications about AE are received and filed.			
13. The corrective and preventive actions (safety CAPA) generated by the audits and inspections are carried out.			
14. The corrective and preventive actions (safety CAPA) generated by monitoring visits are carried out.			
15. The result of corrective and preventive actions generated from safety monitoring is documented.			
16. The appearance of an SAE Is notified to the IRB.			
17. The IRB has control of the SAE management on the site.			

*To specify bellow the cause of non-compliance or partial compliance (PC).

**International Conference on Harmonisation. E6 (R1)- Good Clinical Practice: consolidated guidelines, Step 5. May 1996. Available from: https://www.ich.org/fileadmin/Public_Web_Site/ICH_Products/Guidelines/Efficacy/E6/E6_R1_Guideline.pdf
***Council for International Organizations of Medical Sciences. Working Group VI. Geneva: CIOMS, 2005. Available from: https://cioms.ch/wp-content/uploads/2017/01/Mgment_Safety_Info.pdf

Checklist for assessing adherence to the current regulatory requirements for safety information contained in the essential documents.

Investigator's Brochure

Safety aspects that should appear in the document	Yes	No	NA
1. In case of a new indication, there is a specific manual for her.			
2. It has been updated at least once a year.			
3. Updated if relevant information appeared.			
4. Effects Section in human-safety and efficacy section (safety results in previous trials summarized in tables of events by indication or subgroups of treatment and discussion of differences).			
5. Human Effects Section - safety and efficacy section (Description of possible risks and unanticipated problems).			
6. Summary section of data and guide for the investigator (Description of possible risks and adverse reactions).			
7. Summary section of data and guide for the investigator (guidelines for the recognition and treatment of an overdose and adverse reactions).			

NA: Not applicable.

Research protocol

Safety aspects that should appear in the document	Yes	No	NA
1. In Background: Summary of Potential Risks and Potential Benefits Known to Humans.			
2. In EC design: description of criteria for stopping any part of the trial, the complete trial or withdrawal of a subject from the trial (related to the occurrence of any serious adverse event related to the product.			
3. In the section on adverse events: A list of adverse events that have been most frequently reported in previous clinical trials.			
4. In the section of adverse events: Description of the actions to be taken to detect the occurrence of adverse events.			
5. In the section of adverse events: Description of the algorithm to be used for the classification of the causality of adverse events.			
6. In terms of adverse events: Clinical behavior to follow in the event of an adverse event.			
7. In terms of adverse events: Regulatory behavior to follow in the face of a serious and/or unexpected event.			
8. In the heading of adverse events: Preparation of the personnel who will attend the clinical trial patient to an adverse event.			
9. In the section on adverse events: Definition of serious and unexpected adverse events.			
10. Under heading of adverse events: Data of personnel to notify at the			
11. Minimum or indispensable information required for the expedited report.			

12. In the safety assessment topic: Methods and timetable for evaluating, collecting and analyzing safety parameters.			
13. In the safety assessment topic: Procedures for recording and reporting adverse events and intercurrent illnesses, and reporting on them.			
14. In the safety assessment topic: Type and duration of follow-up of subjects after adverse events.			

NA: Not applicable.

Informed Consent Form

Safety aspects that should appear in the document	Y	No	NA
1. Risks reasonably foreseeable for the subject (description of more frequent adverse events according to previous experiences).			
2. Need to take contraceptive measures (to prevent possible risks of alterations in the reproductive system, damage to the embryo, fetus, or child) if appropriate.			
3. Potential risks and benefits of alternative procedures or treatments.			
4. Compensation and / or treatment available in case of injury caused by the study.			
5. Person to contact in case of damages related to the trial.			

NA: Not applicable.

Section 2:

EXPERT SELECTION QUESTIONNAIRE

Dear professional,

Hereby we are inviting you to collaborate as an Expert, in an investigation whose objective is the validation of a safety management system* during the clinical development of the Center of Molecular Immunology (CIM) products.
For the selection of experts, the coefficient of competence of all invited professionals will be determined, and the panel will be composed of professionals with a medium to high.
Please complete the data and answer the questions below:

Name(s) and surname(s):
College degree:
Scientific grade:
Teaching category:
Years of experience in clinical trial activity:
The role that has played in relation to this activity:
1. Mark with a cross (X) on a growing scale from 1 to 10, the value that corresponds to the degree of knowledge or information you have on the subject to study (Safety management during the conduct of clinical trials):

1	2	3	4	5	6	7	8	9	10

2. Taking into account the high, medium and low criteria, rate the influence that each aspect of those listed in the table below has had, in the level of argumentation or rationale you have on the subject in question.

**Sources of argument**	**Level of argument**		
	**High**	**Medium**	**Level**
1- Theoretical analyzes linked to safety management during the conduction of clinical trials.			
2- Experience gained in practice regarding security management.			
3- Revised works of national authors on the subject.			
4- Revised works by international authors on the subject.			
5- Knowledge of the state of security management abroad.			
6- Your intuition			

Thank you very much in advance for your response. Wait for notification.

MSc. Yaimarelis Saumell Nápoles
Principal investigator of the Project

List of experts who formed the panel after the selection process

- ✓ Dr. Judith Daysi Cartaya lópez. Doctor. Master in Pharmacology. Member of the Clinical Trials Authorization Group. Department of Investigation and Evaluation of Drugs and Biologics. Center for State Control of the Quality of Medicines (CECMED), Cuba.
- ✓ Dr. Lídice Fernández Gutiérrez. Doctor. Master in Pharmacology. Staff member of the Clinical Trials Authorization Group. Department of Investigation and Evaluation of Drugs and Biologics. Center for State Control of the Quality of Medicines (CECMED), Cuba.
- ✓ MSc. Eric Chong. Master in Biotechnology. Head of the Regulatory Affairs Department of the Molecular Immunology Center, Cuba.
- ✓ Lic. Lázara Castillo Díaz. Specialist at the Regulatory Affairs Department of the Molecular Immunology Center, Cuba.
- ✓ MSc. Leticia Cabrera Degree in Nursing. Clinical Research Manager. Center of Molecular Immunology. Havana, Cuba. Diploma in Clinical Safety.

- ✓ Lic. Meylan Cepeda Portales. Degree in Nursing. Clinical monitor and representative of the Center for Molecular Immunology in Villa Clara province, Cuba. Diploma in Clinical Safety.
- ✓ MSc. Geydi Lorenzo Monteagudo. Degree in Pharmacy. Clinical Research Manager. Center of Molecular Immunology. Havana, Cuba. Diplomada in Clinical Safety.
- ✓ MSc. Ana Rosa Valls Hung. Degree in Pharmacy. Clinical Research Manager. Center of Molecular Immunology. Havana, Cuba. Diploma in Clinical Safety.
- ✓ Lic. Delmis Mayra Batista Footwear. Degree in Nursing. Clinical monitor of the Center for Molecular Immunology in Holguín province, Cuba.
- ✓ Lic. Dianne Griñán Semaná. Provincial Coordinator of Clinical Trials of Santiago de Cuba Province and clinical monitor.

Section 3:

Validation guide based on expert judgment

Annex you will find eight instruments. You must make an exhaustive assessment of each of them with your knowledge. The items or questions were elaborated from the regulatory requirements in force on the subject, fundamentally from the Guideline E6: Good Clinical Practice of the International Conference on Harmonization (ICH).
You can assign to each aspect a single rating range, and suggest modifications or any questions you would add. Your judgment will serve to evaluate the work and improve it. Appropriateness was designated as a scale from 1 to 5 (1 represents a maximum full disagreement while 5, shows full agreement), and the response categories were described by using the following linguistic qualifiers:
5) Very adequate (VA): It is the item or question wish optimally reflects the theme, concept or specific content that it tries to measure. It can provide the total grasping of that content for clarity in writing (comprehension) and adequacy of the response options (for knowledge and satisfaction surveys).
4) Quite adequate (QA): It is the item or question that express the theme in a quite high degree, content or specific item content that it tries to measure. Although it requires some drafting modification is an adequate response option and provide a high level acquisition of the content.

3) Adequate (A): It is the item or question that takes into account an important part of the qualities of the subject or specific item that it tries to measure. Although it can be improved by the experts modifying some part of the text.

2) Little adequate (LA): The item or question that reflects a low level of adjusted theme. The concept or specific content that it tries to measure must be considered. This category involves a low adequate level of the evaluated subject. The text may be modified by the expert point of view.

1) Inadequate (I): It is an aspect with marked limitations and contradictions. It is that not allowed express the essential qualities of the concept or specific content item. It is intended to measure, but ideas are not stated correctly. It implies the failure of grasping or understanding the element in question and, consequently, brings about the deletion of the item.

Title of the Instrument:

____

____

**Item or question**	**Very Adequate (VA)**	**Quite Adequate (QA)**	**Adequate (A)**	**Little Adequate (LA):**	**Inadequate (I)**
**1.**					
**2.**					
**3.**					
• .					
•					
• .					
**n**					

**Modifications proposed:**
**Questions you would add:**

Section 4

Table S2. Application of Delphi Method: A Survey of Professional's Knowledge at the Investigational Sites

Theme of the questions	Absolute Frequencies			Cumulative Absolute			Cumulative Relative Frequencies			Cutoffs and Resume						
	VA	QA	A	VA	QA	A	VA	QA	A	VA	QA	A	S	AR	N-AR	Categ
1. Definition of adverse event.	10	0	0	10	10	10	1	1	1	3.49	3.49	3.49	10.47	3.49	-1.32	MA
2. Responsible for the detection of AE	7	2	1	7	9	10	0.7	0.9	1	0.52	1.28	3.49	5.30	1.77	0.41	MA
3. Types of AE	8	2	0	8	10	10	0.8	1	1	0.84	3.49	3.49	7.82	2.61	-0.43	MA
4. Sources of AE	10	0	0	10	10	10	1	1	1	3.49	3.49	3.49	10.47	3.49	-1.32	MA
5. Definition of serious adverse event.	10	0	0	10	10	10	1	1	1	3.49	3.49	3.49	10.47	3.49	-1.32	MA
6. Treatment of AE	10	0	0	10	10	10	1	1	1	3.49	3.49	3.49	10.47	3.49	-1.32	MA
7. Interruption due to AE	10	0	0	10	10	10	1	1	1	3.49	3.49	3.49	10.47	3.49	-1.32	MA
8. Severity / intensity of AE	9	1	0	9	10	10	0.9	1	1	1.28	3.49	3.49	8.26	2.75	-0.58	MA
9. Seriousness / severity of AE	10	0	0	10	10	10	1	1	1	3.49	3.49	3.49	10.47	3.49	-1.32	MA
10. Algorithms of causality	10	0	0	10	10	10	1	1	1	3.49	3.49	3.49	6.98	3.49	-1.32	MA
11. Previous knowledge of the AE	7	3	0	7	10	10	0.7	1	1	0.52	3.49	3.49	7.50	2.50	-0.33	MA
12. Safety Information to IRB	9	1	0	9	10	10	0.9	1	1	1.28	3.49	3.49	8.26	2.75	-0.58	MA
13. Rupture of blinding by AE	10	0	0	10	10	10	1	1	1	3.49	3.49	3.49	10.47	3.49	-1.32	MA
14. Responsible for identifying the EA	8	2	0	8	10	10	0.8	1	1	0.84	3.49	3.49	7.82	2.61	-0.43	MA
15. Concept of unexpected event	8	2	0	8	10	10	0.9	1	1	1.28	3.49	3.49	8.26	2.75	-0.58	MA
16. Traceability classification AE	9	1	0	9	10	10	0.9	1	1	1.28	3.49	3.49	8.26	2.75	-0.58	MA
17. AE that require notification.	9	1	0	9	10	10	0.9	1	1	1.28	3.49	3.49	8.26	2.75	-0.58	MA
18. Type of AE requiring ER	9	1	0	9	10	10	0.9	1	1	1.28	3.49	3.49	8.26	2.75	-0.58	MA
19. Deadlines for delivery ER	8	2	0	8	10	10	0.8	1	1	0.84	3.49	3.49	7.82	2.61	-0.43	MA
20. Traceability of ER from the site	8	2	0	8	10	10	0.8	1	1	0.84	3.49	3.49	7.82	2.61	-0.43	MA
Cutoffs by category (total average)										1.92	3.38	3.49	173.93	N=173.9/(20x4)=2.21		

Abbreviations: AE, Adverse Event; SAE, Serious adverse event; RA, Regulatory Agency; VA, Very adequate; QA, Quite adequate;

A, Adequate; ER, Expedited Report; IRB, Institutional Review Board; S, Sum; AR, Average row; N, limit value.

Table S3. Results of Delphi Method: Satisfaction Survey of Internal and External Clients

Theme of the Items	Absolute Frequencies				Cumulative Absolute				Cumulative Relative Frequencies				Cutoffs and Resume							
	VA	QA	A	LA	VA	QA	A	LA	VA	QA	A	LA	VA	QA	A	LA	S	AR	N-AR	Categ
1. The term used for name the AE in medical records, ER or notification is standardized.	7	2	0	1	7	9	9	10	0.7	0.9	0.9	1	0.52	1.28	1.28	3.49	6.58	2.62	-0.21	MA
2. The description of AE in medical records. models of notification or ER is appropriate.	5	5	0	0	5	10	10	10	0.5	1	1	1	0.00	3.49	3.49	3.49	10.47	2.94	-0.53	MA
3. The classification of AE according to the intensity complies with the requirements of the protocols.	9	1	0	0	9	10	10	10	0.9	1	1	1	1.28	3.49	3.49	3.49	11.75	2.94	-0.53	MA
4. The categories used in the classification of the AE is according to the causality algorithms proposed in the protocol.	9	1	0	0	9	10	10	10	0.9	1	1	1	1.28	3.49	3.49	3.49	11.75	3.49	-1.08	MA
5. The reporting of AE in the notification models is complete.	10	0	0	0	10	10	10	10	1	1	1	1	3.49	3.49	3.49	3.49	13.96	3.49	-1.08	MA
6. The AE information in the notification models is timely (arrives in the set time).	10	0	0	0	10	10	10	10	1	1	1	1	3.49	3.49	3.49	3.49	13.96	3.49	-1.08	MA
7. The report of AE in the ER models is complete.	10	0	0	0	10	10	10	10	1	1	1	1	3.49	3.49	3.49	3.49	13.96	3.49	-1.08	MA
8. The adverse event information models ER is timely (arrives in the set time).	10	0	0	0	10	10	10	10	1	1	1	1	3.49	3.49	3.49	3.49	13.96	3.49	-1.08	MA
Cutoffs by category (total average)													2.13	3.21	3.21	3.49	96.39	N=96.39/(8x5)= 2.41		

Abbreviations: AE: Adverse Event: SAE: Serious adverse event RA: Regulatory Agency VA: Very adequate QA: Quite adequate
A: Adequate LA: Little adequate ER: Expedited Report S: Sum AR: Average row N- limit value CR*:* Clinical record

Table S4. Results of Delphi Method: Patient's Satisfaction Survey

Theme of the items	Absolute Frequencies				Cumulative Absolute				Cumulative Relative Frequencies				Cutoffs and Resume							
	VA	QA	A	LA	VA	QA	A	LA	VA	QA	A	LA	VA	QA	A	LA	S	AR	N-AR	Categ
1. The attention was immediately to the appearance of the AE.	10	0	0	0	10	10	10	10	1	1	1	1	3.49	3.49	3.49	3.49	13.96	3.49	-1.34	MA
2. The doctor explained the consequences of the event for your current health status.	8	1	0	1	8	9	9	10	0.8	0.9	0.9	1	0.84	1.28	1.28	3.49	6.89	1.72	0.43	MA
3. The classification of AE according to the intensity complies with the requirements of the protocols.	10	0	0	0	10	10	10	10	1	1	1	1	3.49	3.49	3.49	3.49	13.96	3.49	-1.34	MA
4. They explained the consequences of the AE to continue in the study and receive the product.<br>Algorithms proposed in the protocol.	8	1	1	0	8	9	10	10	0.8	0.9	1	1	0.84	1.28	3.49	3.49	9.10	2.28	-0.13	MA
5. You are satisfied with the care received when appeared and adverse event that required treatment.	6	3	1	0	6	9	10	10	0.6	0.9	1	1	0.25	1.28	3.49	3.49	8.51	2.13	0.02	MA
6. You were informed of the reactions that may occur after administration even before the first dose of the product.	7	1	0	2	7	8	8	10	0.7	0.8	0.8	1	0.52	0.84	0.84	3.49	5.70	1.42	0.73	MA
7. Even before the first dose of the product you know the person to contact if you a life-threatening AE or the required/prolonged hospitalization.	10	0	0	0	10	10	10	10	1	1	1	1	3.49	3.49	3.49	3.49	13.96	3.49	-1.34	MA
8. You are satisfied with the information received regarding the safety of the product under investigation.	10	0	0	0	10	10	10	10	1	1	1	1	3.49	3.49	3.49	3.49	13.96	3.49	-1.34	MA
Cutoffs by category (total average)													2.13	3.21	3.21	3.49	96.39	N=86.05/(8x5)= 2.15		

Abbreviations: AE, Adverse Event; SAE, Serious adverse event; RA, Regulatory Agency; VA, Very adequate; QA, Quite adequate; A, Adequate; LA, Little adequate; ER, Expedited Report; S, Sum; AR, Average row; N, limit value.

Table S5. Results of Delphi Method: Checklists for Evaluate Safety Management Infrastructure at the Sponsoring Center

Theme of the Items	Absolute Frequencies			Cumulative Absolute			Cumulative Relative			Cutoffs and Resume						
	VA	QA	A	VA	QA	A	VA	QA	A	VA	QA	A	S	AR	N-AR	Categ
1. There is a database for managing and archiving the safety information.	10	0	0	10	10	10	1	1	1	3.49	3.49	3.49	10.47	3.49	-0.94	MA
2. There is a register for controlling the input and output notification / ER form in the site.	10	0	0	10	10	10	1	1	1	3.49	3.49	3.49	10.47	3.49	-0.94	MA
3. There is a SOP or instruction to guide the global management of safety data.	10	0	0	10	10	10	1	1	1	3.49	3.49	3.49	10.47	3.49	-0.94	MA
4. Clinical safety events are analyzed (identification, coding, quantification and evaluation) as is established by pharmacovigilance.	10	0	0	10	10	10	1	1	1	3.49	3.49	3.49	10.47	3.49	-0.94	MA
5. The number of notification or expedited report is controlled by investigational product or indication.	10	0	0	10	10	10	1	1	1	3.49	3.49	3.49	10.47	3.49	-0.94	MA
6. The date of start and end of the AE is controlled.	10	0	0	10	10	10	1	1	1	3.49	3.49	3.49	10.47	3.49	-0.94	MA
7. The dates of receipt the notification/ ER of an AE are controlled.	10	0	0	10	10	10	1	1	1	3.49	3.49	3.49	10.47	3.49	-0.94	MA
8. The causality of case reports and series of individual cases are evaluated.	10	0	0	10	10	10	1	1	1	3.49	3.49	3.49	10.47	3.49	-0.94	MA
9. Criteria for assessing the quality of notifications or accelerated reports are defined.	10	0	0	10	10	10	1	1	1	3.49	3.49	3.49	10.47	3.49	-0.94	MA
10. The evidence of communications between researchers and promoters about adverse events are archived.	10	0	0	10	10	10	1	1	1	3.49	3.49	3.49	10.47	3.49	-0.94	MA
11. The results of safety corrective and preventive actions (CAPA) generated in monitoring. audits and inspections reports) are filed, followed and disseminated.	10	0	0	10	10	10	1	1	1	3.49	3.49	3.49	10.47	3.49	-0.94	MA
12. The corrective and preventive actions generated of the safety CAPA are documented.	10	0	0	10	10	10	1	1	1	3.49	3.49	3.49	10.47	3.49	-0.94	MA

13. The inter-institutional or informational communications about safety are sent to the stakeholders and are archived.	10	0	0	10	10	10	1	1	1	3.49	3.49	3.49	10.47	3.49	-0.94	MA
14. The responses to requests for notifiers and the regulatory agency are managed and are filed.	10	0	0	10	10	10	1	1	1	3.49	3.49	3.49	10.47	3.49	-0.94	MA
15. The changes in benefit/risk for investigational product are evaluated and are communicated to researchers.	7	1	2	7	8	10	0.7	0.8	1	0.52	0.84	3.49	4.85	1.62	0.93	MA
16. Training and counseling processes about safety are coordinated for the members of the research teams.	10	0	0	10	10	10	1	1	1	3.49	3.49	3.49	10.47	3.49	-0.94	MA
17. The dates of the Development Safety Update Report (DSUR) actualization are controlled.	10	0	0	10	10	10	1	1	1	3.49	3.49	3.49	10.47	3.49	-0.94	MA
18. The date of update safety data in Investigator Brochure, Protocol and Informed Consent, are controlled.	10	0	0	10	10	10	1	1	1	3.49	3.49	3.49	10.47	3.49	-0.94	MA
19. The risk management plans for the investigational product are elaborated.	10	0	0	10	10	10	1	1	1	3.49	3.49	3.49	10.47	3.49	-0.94	MA
20. The internal audits to safety management are performed.	10	0	0	10	10	10	1	1	1	3.49	3.49	3.49	10.47	3.49	-0.94	MA
Cutoffs by category (total average)									3.34	3.36	3.49	203.78	N=203.78/(20x4)=2.55			

Abbreviations: AE, Adverse Event; SAE, Serious adverse event; RA, Regulatory Agency (CECMED); VA, Very adequate; QA, Quite adequate; A, Adequate; ER, Expedited Report; S, Sum; AR, Average row; N, limit value; PSUR, Periodic Safety Update Report.

Table S6. Results of Delphi Method for the Checklists for Evaluate Safety Management Infrastructure in the Investigational Sites

Theme of the items	Absolute frequencies			Cumulative absolute			Cumulative relative			Cut offs and resume						
	VA	QA	A	VA	QA	A	VA	QA	A	VA	QA	A	S	A	N-AR	Categ
18. They have a crash cart during the administration of a new investigational product.	9	1	0	9	10	10	0.9	1	1	1.28	3.49	3.49	8.26	2.75	-0.74	MA
19. The use of a truck stop for clinical research is documented.	10	0	0	10	10	10	1	1	1	3.49	3.49	3.49	10.47	3.49	-1.48	MA
20. They have a stock of medicines for emergencies during the clinical trials.	9	1	0	9	10	10	0.9	1	1	1.28	3.49	3.49	8.26	2.75	-0.74	MA
21. The use of emergency stock for clinical research is documented.	10	0	0	10	10	10	1	1	1	3.49	3.49	3.49	10.47	3.49	-1.48	MA
22. There is a Standard operating procedure or instruction for management safety data at the investigational site.	10	0	0	10	10	10	1	1	1	3.49	3.49	3.49	10.47	3.49	-1.48	MA
23. All serious adverse events are analyzed.	8	1	1	8	10	10	0.8	1	1	0.84	3.49	3.49	7.82	2.61	-0.60	MA
24. The number of notifications and ER is controlled by investigational product or indication.	10	0	0	10	10	10	1	1	1	3.49	3.49	3.49	10.47	3.49	-1.48	MA
25. The dates of start and end of the AE are controlled.	8	1	1	8	9	10	0.8	0.9	1	0.84	1.28	3.49	5.61	1.87	0.14	MA
26. The date of shipment of AE notification and ER are controlled.	10	0	0	10	10	10	1	1	1	3.49	3.49	3.49	10.47	3.49	-1.48	MA
27. If necessary. the promoter is alert when signals are generated and causality series are evaluated.	10	0	0	10	10	10	1	1	1	3.49	3.49	3.49	10.47	3.49	-1.48	MA
28. The quality of notifications and ER that are sent to the promoter are guaranteed.	10	0	0	10	10	10	1	1	1	3.49	3.49	3.49	10.47	3.49	-1.48	MA
29. All the communications about AE are received and filed.	10	0	0	10	10	10	1	1	1	3.49	3.49	3.49	10.47	3.49	-1.48	MA
30. The corrective and preventive actions (safety CAPA) generated by the audits and inspections are carried out.	10	0	0	10	10	10	1	1	1	3.49	3.49	3.49	10.47	3.49	-1.48	MA

31. The corrective and preventive actions (safety CAPA) generated by monitoring visits are carried out.	10	0	0	10	10	10	1	1	1	3.49	3.49	3.49	10.47	3.49	-1.48	MA
32. The result of corrective and preventive actions generated from safety monitoring is documented.	10	0	0	10	10	10	1	1	1	0.52	0.84	3.49	4.85	1.62	0.39	MA
33. The appearance of an SAE Is notified to the IRB.	10	0	0	10	10	10	1	1	1	3.49	3.49	3.49	10.47	3.49	-1.48	MA
34. The IRB has control of the SAE management on the site.	10	0	0	10	10	10	1	1	1	3.49	3.49	3.49	10.47	3.49	-1.48	MA
Cutoffs by category (total average)										2.74	3.20	3.49	160.44	N=160.44/(20x4)=2.01		

Abbreviations: AE, Adverse Event; SAE, Serious adverse event; RA, Regulatory Agency; VA, Very adequate; QA, Quite adequate; A, Adequate; ER, Expedited Repo.
